# Social inequalities in access to cancer screening and early detection: A population-based study in the city of São Paulo, Brazil

**DOI:** 10.1016/j.clinsp.2022.100160

**Published:** 2023-01-19

**Authors:** Edige Felipe de Sousa Santos, Camila Nascimento Monteiro, Diama Bhadra Vale, Marília Louvison, Moisés Goldbaum, Chester Luiz Galvão Cesar, Marilisa Berti de Azevedo Barros

**Affiliations:** a Hospital Sírio-Libanês, São Paulo, SP, Brazil; b Universidade Estadual de Campinas (UNICAMP), Campinas, SP, Brazil; c Faculdade de Saúde Pública, Universidade de São Paulo (USP), São Paulo, SP, Brazil; d Faculdade de Medicina, Universidade de São Paulo (USP), São Paulo, SP, Brazil

**Keywords:** Socioeconomic inequalities, Secondary prevention, Mass screening, Early detection of cancer, Uterine cervical neoplasms, Breast neoplasms, Prostatic neoplasms

## Abstract

**Objective:**

This study monitors trends in access to cancer screening, focusing on mammography, Papanicolaou (Pap smear), and Prostate-Specific Antigen (PSA), assessing the magnitude of inequality in the city of São Paulo from 2003 to 2015 according to education level.

**Method:**

This is a cross-sectional population-based study conducted with data from the 2003, 2008, and 2015 editions of the Health Survey of the City of São Paulo (ISA-Capital). Outcome variables were the proportion of mammography, Papanicolaou (Pap smear), and Prostate-Specific Antigen (PSA) tests according to the protocols. Inequality was measured by education level according to years of study. For static analysis, Poisson regression was used to estimate proportion ratios.

**Results:**

The proportion of Pap smears remained stationary at a high level (>89%) throughout the study period, while access to mammography and PSA tests significantly increased in the 2003‒2015 period. The present results indicate inequalities in access to cancer screening due to education, and being more expressive for mammography and PSA tests. However, this inequality significantly decreased over the period analyzed comparing the most educated individuals with those with the lowest educational level. In addition, an increase in the proportion of tests performed in the Brazilian Unified Health System was identified, especially for mammography and PSA tests, in the period 2003‒2015.

**Conclusions:**

The inequalities observed in the access to preventive exams were influenced by the level of education. The offer of exams was expanded, more significantly for mammography and PSA, especially among the less educated group.

## Highlights

• The proportion of Pap smears remained at a high level (>89%) from 2003 to 2015 in São Paulo.
• The offer of exams was expanded, more significantly for mammography and PSA, especially among the less educated group.
• Were identified inequalities in access to cancer screening due to education, being more expressive for mammography and PSA tests.

## Introduction

With rapidly increasing incidence and mortality rates, cancer is a global health problem that imposes barriers to prolonging life expectancy and causes premature death in most countries, including Brazil.^1^^,^^2^ Breast and prostate cancers are the most prevalent types of cancer in all regions of Brazil – except in the North region, where the incidence of cervical cancer is similar to that of breast cancer. According to 2020 estimates from the National Cancer Institute, there will be 625,000 new cases of cancer each year in the country.^2^ In 2018, more than 107,000 Brazilian women died from cancer, including 16% as a result of breast cancer and 6% from cervical cancer. In Brazil, after skin cancer, prostate cancer is the one with the highest incidence, being the fourth leading cause of death from neoplasms in men.^2^

The Ministry of Health recommends the use of mammograms and Pap Smears for screening for breast and cervical cancers.^3^^,^^4^ Aiming to reduce morbidity and mortality due to breast cancer in Brazil, the National Protocol intends to improve access to and coverage for mammograms, reaching 60% of the target population: women aged 50‒69 years, undergoing the test every two years.^2^^,^^5^

Women aged 25‒64 years (the target population) should undergo Pap smear screenings for cervical cancer every three years.^3^ Pap smears are well accepted by women and widely offered in primary healthcare units in Brazil, so the country expects to achieve 85% coverage by 2022.^6^^,^^7^ However, programs aimed at promoting screening and early detection of breast and cervical cancers in Brazil sometimes do not reach the entire target population. In the period from 2016 to 2021, there is a stable supply of cervical cytopathological exams from SUS in Brazil, with a decline at the end of the period. As a consequence of the COVID-19 pandemic, there was a drop in exams in 2020. In 2021 there is an increase in the number of exams compared to 2020, but still lower to reliable levels in the years before the pandemic.^8^^,^^9^

Prostate cancer is one of the highest incidence types of cancer among Brazilian men. Data from the National Cancer Institute show an estimated 65,840 new cases in 2020 and 15,576 deaths due to the disease in 2018.^2^ Digital Rectal Examinations (DRE) and Prostatic Specific Antigen (PSA) tests are common techniques employed in clinical practice for prostate cancer screening. However, the Ministry of Health does not recommend population screening.^10^

A population-based health survey of the city of São Paulo (ISA-Capital) analyzed preventive practices for screening and early detection of cancer to assess the magnitude of social inequalities and monitor trends in inequalities.^11^, ^12^, ^13^ Despite being the most populous megalopolis in Brazil and having above-average socioeconomic and health indicators,^14^ São Paulo accounts for one of the highest inequality indices in Latin America, affecting access to health services and the performance of preventive practices.^15^ Despite recommendations for periodic screening through mammograms and Pap smears, studies in the national and international scenarios have identified a series of sociodemographic and socioeconomic factors limiting access to tests, indicating the existence of social inequalities in access to cancer prevention and detection practices.^11^^,^^16^, ^17^, ^18^, ^19^ Thus, this study aimed to assess changes in the frequency of preventive cancer detection practices, and to assess the evolution of inequality in access to Pap smears, Mammography and PSA, according to education level, in the population residing in the urban area. of the city of São Paulo, in the period 2003‒2015.

## Methods

### Study design

The ISA-Capital is a cross-sectional population-based survey conducted by means of household interviews with the urban population of the city of São Paulo in the years 2003, 2008, and 2015, to analyze health and living conditions, including preventive practices.^12^ The survey was supported by the Municipal Health Secretariat of São Paulo, in partnership with the School of Public Health of the University of São Paulo.

In total, 3,357 individuals were interviewed by the ISA-Capital in 2003; 3,271 in 2008; and 4,043 in 2015. Sampling was performed by a two-stage clustering, stratified into census sectors and households. Further details on the sampling methods adopted in the ISA-Capital editions can be seen on the ISA-Capital website (https://www.prefeitura.sp.gov.br/cidade/secretarias/saude/epidemiologia_e_informacao/isacapitalsp/) and in previous publications.^20^, ^21^, ^22^

### Study population

All men and women eligible to answer the section of the ISA-Capital questionnaire related to preventive practices were included in the study, considering Pap smear tests in the previous three years for women aged 25‒64; mammograms in the previous two years for women aged 50‒69 years old; and PSA at least once in their lives for men older than 40 years. With that, 1,125 individuals from the 2003 survey were included; 1,410 from 2008; and 2,234 from 2015.

### Variables

The study population was characterized based on the following socioeconomic and demographic variables: sex (male and female), age (women 25‒39, 40‒59, 60‒64 years and men 40‒49 years, 50‒59, and 60 years or more), self-reported ethnicity (white and non-white ‒ the latter including brown and black), income (monthly per capita family income: ≤ 1 national minimum wage, > 1.1 to 4.99, ≥ 5), education level in years (≤ 7, 8‒11, 12 or more), and access to private healthcare (yes or no). Although 6,018,886 million of the population of São Paulo are covered by private health services, which corresponds to 50.3% of beneficiaries of private health plans, health in Brazil is universal and free.^23^ The studied variables were the proportion of Pap smears, mammography, and PSA in the target population. Blank information was considered missing and was removed from the analysis

### Statistical analysis

In the weighted sample, individuals were initially characterized according to sex and education level. To verify the magnitude of inequality in the proportion of diagnostic tests, proportion ratios and 95% Confidence Intervals (95% CI) were estimated by Poisson regression according to education level (≤ 7 years, 8‒11 years, and 12 years or more)

To assess differences in the magnitude of inequality between the study periods ‒ “2003‒2008”, “2008‒2015” and “2003‒2015” ‒ and to verify increases in the proportion of practices between the study periods, the overlap of the 95% Confidence Intervals (95% CI) was considered or disregarded. Regression models were adjusted according to age. Was considered a descriptive level of 0.05 for the Wald test.

All analyzes were performed using the Survey module of the Stata® 14.0 software (https://www.stata.com), taking into account the complex effects of the study design and the effect of stratification and embedding the different observation weights.

This study was approved by the Ethics Committee of the School of Public Health of the University of São Paulo (protocol: 719,661/2014) The contact of the Ethics Committee is: coep@fsp.usp.br. Written informed consent was obtained from all participants for the study.

## Results

In the most recent period (2015), 59.6% of women aged 25‒69 were white, 43.9% had an education level of 8‒11 years, 58.1% had an income ≤1 minimum wage and 45.1% had a health plan. As for men aged 40 years and over, 56.1% were white, 38.2% had between 8‒11 years of schooling, 52.1% had an income ≤1 minimum wage and 43.7% had possession of a health plan. The characterization of the study population, according to age group, education, and access to private healthcare is shown in Table 1.

**Table 1:** Sample description according to demographic and socioeconomic factors ‒ São Paulo, 2003, 2008, and 2015.

Women						
	2003		2008		2015	
Variables	n^a^ (563)	%^b^	n (836)	%	n (1412)	%
**Age Group**						
25 to 39 years	181	46.7	278	42.9	507	41.8
40 to 59 years	152	41.9	299	46.3	555	44.1
60 to 69 years	230	11.5	259	10.8	350	14.1
**Years of study**^c^						
Less than 8 years	327	43.0	360	28.9	412	23.2
8 to 11 years	146	33.5	338	47.1	613	43.9
More than 11 years	79	23.5	137	24.1	377	32.9
**Private healthcare**						
No	‒	‒	470	50.8	825	54.9
Yes	‒	‒	366	49.2	587	45.1

Men						
	2003		2008		2015	
Variables	n (562)	%	n (574)	%	n (822)	%
**Age Group**						
40 to 49 years	73	36.2	131	43.8	235	38.5
50 to 59 years	68	33.7	87	27.9	200	30.1
60 years or older	421	30.1	356	28.3	387	31.3
**Years of study**^d^						
Less than 8 years	361	45.9	320	38.5	359	36.2
8 to 11 years	116	27.0	179	39.4	297	38.2
More than 11 years	75	27.0	74	22.0	163	25.6
**Private healthcare**^e^						
No	‒	‒	318	52.4	508	56.3
Yes	‒	‒	256	47.6	312	43.7

Source: ISA-Capital, 2003, 2008, 2015.

a Number of individuals in the unweighted sample.

b Proportions calculated under weighting.

c 11 (1.9%) missing (2003), 1 (0.1%) missing (2008), 10 (0.7%) missing (2015).

d 10 (1.8%) missing (2003), 1 (0.2%) missing (2008), 3 (0.4%) missing (2015).

e – (2003), no missing (2008), 2 (0.4%) missing (2015).

In the most recent period (ISA-Capital 2015), 89.6% of the women aged 25‒64 years reported having had a Pap smear in the previous three years. Of those aged 50‒64 years, 73.8% had a mammogram in the previous two years. As for men, 63.2% reported having undergone a PSA test at least once in their lives (Table 2). Regarding access to preventive practices for cancer detection, the proportion of having had a Pap smear in the last 3 years remained stationary at a high level (> 80%) in the period 2003‒2015, while there was a significant increase in access to mammograms between 2003‒2015 for women with < 12 years of schooling, and a significant increase in access to PSA testing between 2003‒2015 for men regardless of education level (Table 2).

**Table 2:** Proportion of tests according to complete years of study, ISA-Capital, São Paulo, 2003, 2008 and 2015.

	2003	2008	2015
Years of study	Proportion^a^ % (95% CI)^b^	Proportion^a^ % (95% CI)^b^	Proportion^a^ % (95% CI)^b^
**Pap smear**^c^			
Less than 8 years	88.5 (82.8, 92.5)	83.1 (76.2, 88.4)	82.9 (77.6, 87.2)
8 to 11 years	92.7 (86.0, 96.3)	91.8 (87.5, 94.7)	89.5 (86.5, 91.9)
More than 11 years	99.1 (93.4, 99.9)	95.4 (88.9, 98.2)	93.9 (90.7, 96.0)
Total	92.4 (89.3, 94.6)	90.3 (87.5, 92.6)	89.6 (87.7, 91.3)
**Mammography**^d^			
Less than 8 years	53.8 (42.9, 64.3)	60.3 (50.9, 68.9)	69.2 (62.9, 74.8)
8 to 11 years	45.9 (30.0, 62.8)	79.0 (69.1, 86.3)	73.8 (66.2, 80.2)
More than 11 years	90.4 (78.2, 96.1)	91.6 (76.2, 97.3)	80.1 (72.6, 85.9)
Total	55.6 (46.6, 64.2)	74.3 (68.0, 79.7)	73.8 (69.6, 77.7)
**PSA**^e^			
Less than 8 years	20.5 (14.8, 27.6)	39.5 (31.8, 47.8)	56.4 (50.4, 62.3)
8 to 11 years	24.8 (16.4, 35.7)	45.1 (36.9, 53.5)	58.2 (51.7, 64.5)
More than 11 years	67.4 (51.8, 79.9)	64.8 (48.5, 78.4)	80.1 (72.4, 86.1)
Total	34.6 (28.2, 41.6)	47.3 (41.1, 53.7)	63.2 (59.3, 67.0)

Pap smears, Women aged between 25‒64 years during the last three years; Mammography: Women aged between 50‒69 years during the last two years; PSA, Men aged 40 years or older, at least once in their lifetime. 95% CI, 95% Confidence Interval.

a Proportions (95% CI) calculated under weighting.

b 95% CI, 95% Confidence Interval.

c 4 (0.9%) missing (2003), 1 (0.1%) missing (2008), 7 (0.6%) missing (2015).

d 6 (2.1%) missing (2003), no missing (2008), 2 (0.5%) missing (2015).

e 2 (1.1%) missing (2003), no missing (2008), 3 (0.6%) missing (2015).

The authors evidenced inequalities in access to Pap smears, mammography, and PSA between 2003 and 2015 according to schooling (Table 3). When evaluating the magnitude of the disparity in access to the three preventive practices in 2003, higher inequalities were identified for Mammography (PR = 1.66; 1.32:2.08), and even more for PSA (PR = 3.89; 2.72:5.57), in relation to Pap smears (PR = 1.11; 1.04:1.17), comparing the most educated individuals (12 years and over) with those with the lowest educational level (less than 7 years of study). In addition, inequality in access to Pap smears remained stationary between 2003‒2015. Inequalities for mammography and PSA suffered a significant reduction in the analyzed period, (PR = 1.16; 1.02:1.32) and (PR = 1.48; 1.29;1.69), respectively, in 2015 (Table 3).

**Table 3:** Proportion ratio of Pap smears, Mammography and PSA tests, according to years of study, ISA-Capital - São Paulo, 2003, 2008, and 2015.

	2003	2008	2015
Years of study	PRa a (95% CI)^b^	PRa a (95% CI)^b^	PRa a(95% CI)^b^
**Pap smear**			
Less than 8 years	1	1	1
8 to 11 years	1.04 (0.96,1.12)	1.10 (1.01,1.19)	1.06 (0.99,1.14)
More than 11 years	1.11 (1.04,1.17)	1.14 (1.04,1.25)	1.12 (1.04,1.19)
**Mammography**			
Less than 8 years	1	1	1
8 to 11 years	0.84 (0.55,1.27)	1.28 (1.04,1.56)	1.07 (0.92,1.23)
More than 11 years	1.66 (1.32,2.08)	1.47 (1.20,1.79)	1.16 (1.02,1.32)
**PSA**			
Less than 8 years	1	1	1
8 to 11 years	1.40 (0.84,2.33)	1.40 (1.08,1.83)	1.17 (1.01,1.35)
More than 11 years	3.89 (2.72,5.57)	1.96 (1.48,2.60)	1.48 (1.29,1.69)

Pap smears, Women aged between 25‒64 years during the last three years; Mammography, Women aged between 50‒69 years during the last two years; PSA, Men aged 40 years or older, at least once in their lifetime.

a PRa (95% CI), Adjusted Prevalece Ratio (95% Confidence Interval) calculated under weighting.

b 95% CI, 95% Confidence Interval.

**Fig. 1:**
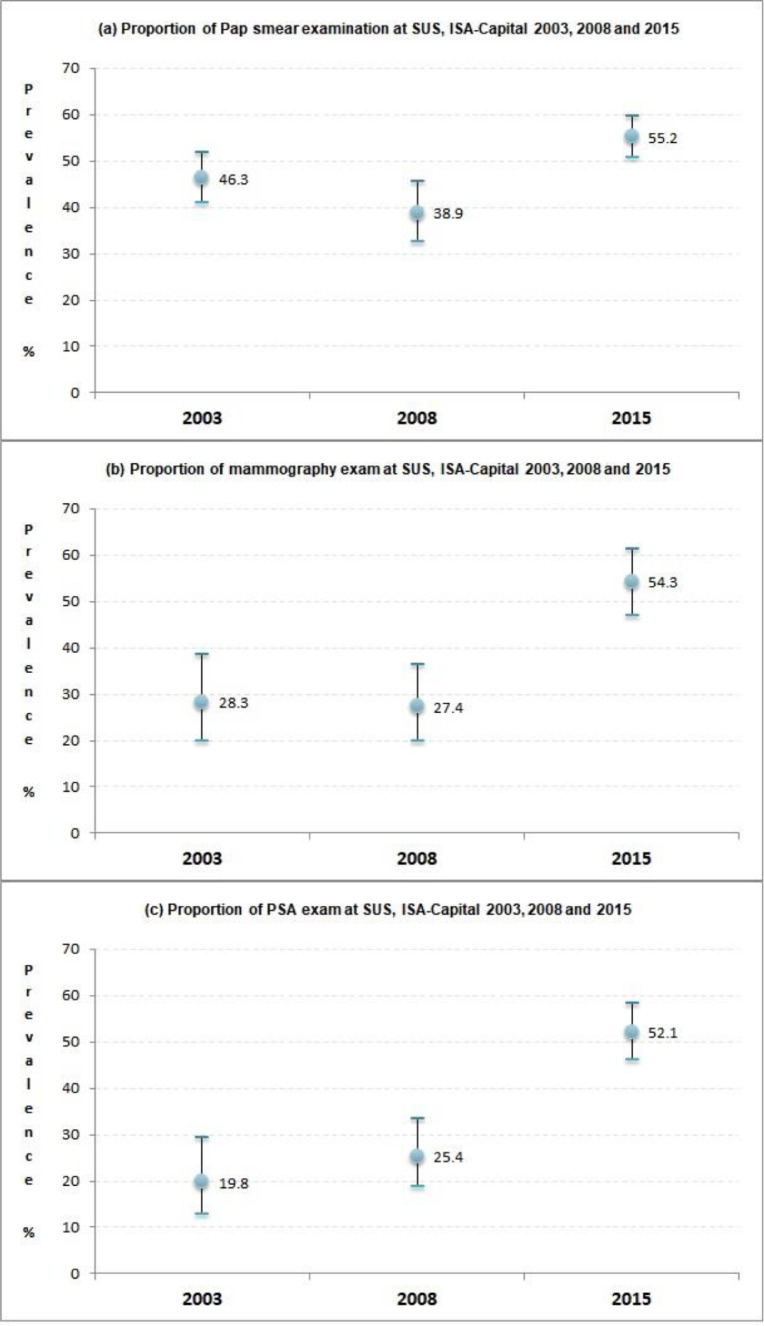
Proportion of tests performed in SUS, ISA-Capital, São Paulo, 2003, 2008 and 2015.

The present results show a significant increase in the coverage of these tests throughout the study period relying exclusively on the public health system, especially mammography and PSA tests, and a decrease of the private sector. Pap smear coverage for this population showed an increase only between 2008‒2015 (Fig. 1).

## Discussion

This study evaluated the changes in the proportion of screening tests and early detection of cancer in the urban population of São Paulo, based on ISA-Capital surveys carried out in 2003, 2008, and 2015. Access to diagnostic tests was greater among more educated individuals, indicating inequalities that persisted until 2015. When comparing the three tests, the authors found greater disparities in access to mammography and Prostate-Specific Antigen (PSA) testing than to Pap smears. In addition, the magnitude of inequalities differed according to the test and over time.

Inequalities in access to mammography and PSA tests according to education level showed a sharp decrease from 2003 to 2015. Despite the challenges in reducing health inequalities in Brazil, the coverage of these diagnostic tests by the public health system significantly increased in recent years. In this sense, the present study data points to a favorable role for the implementation of guiding principles (universality, equity, and integrality) of the public health system in the city of São Paulo.

After almost 20 years of increased coverage, access to Pap smears remained stationary between 2003 and 2015.^24^ Unlike the study conducted in the municipality of Campinas (ISA-Camp), which reported social equity in access to Pap smears,^11^ the present study identified persistent inequalities in access until 2015. However, the magnitude of such disparity in access according to education level was low, and the test coverage was above the target expected by 2022.^8^^,^^9^

There is no consensus between public and private services regarding the recommendations for PSA screening or age group and frequency of mammograms.^2^^,^^4^^,^^25^^,^^26^ It can influence the analysis of social inequalities in health. These results also indicate a significant increase in the proportion of mammography and PSA tests among low-educated women and among men regardless of education level. The magnitude of such disparity gradually decreased over the period of 2003‒2015, especially for PSA tests. However, significant inequalities persist in the most recent period (2015).

Although widely recommended by Medical Societies and the Ministry of Health in Brazil,^5^ coverage rates related to cancer screening and early detection were low for mammography. Public health policies in Brazil depend on funding and, in recent years, there has been a reduction in financial resources devoted to these health policies, which may make it difficult to implement cancer screening policies in the Unified Health System.^27^^,^^28^ Thus, in the most recent period (2015), 61.5% of men reported having undergone a PSA exam at least once in their lives. As for women, 89.6% of those aged 25‒64 years reported having undergone a Pap smear in the last three years and 73.8% of those aged 50‒64 underwent a mammogram in the past two years.

Inequalities in access to mammography and PSA tests were higher than those recorded for access to Pap smears, which not only remained stationary during the study period, but also presented a low magnitude, being 12% higher among women with more than 12 years of education in the year of 2015.

The coverage of mammography and PSA tests by the public health system increased between 2003 and 2015. This fact may be explained by the expanded coverage of Family Health Strategy (FHS) teams in the city of São Paulo, going from 15.9% in 2003 to 26.0% in 2008 and 32.9% in 2015, which expanded the capacity of the public health system for cancer screening and early detection.^29^ The FHS plays a key role in promoting preventive tests and educational actions in health, thus increasing the number of referrals of positive tests to other care levels^30^^,^^31^ and expanding mammography offerings as part of federal and state incentives.^31^ The present results indicate that although a significant number of men now have access to PSA tests (especially in the public health system), access to this diagnostic test implies persistent socioeconomic inequalities until 2015. These results are aligned with those reported by a population-based study conducted with data from the Multicenter Health Survey in the State of São Paulo (ISA-SP) in the period of 2001‒2002, which identified an association between the lack of access to prostate cancer screening tests and individuals with up to 8 years of education.^32^ The authors emphasize that PSA is not a guideline by the Ministry of Health. Moreover, the lower proportion of men accessing these tests may be explained by their overall lower use of or access to health services when compared to women.^33^

Performing a mammography is a guideline of the Brazilian Ministry of Health. The authors verified a decrease in inequalities in access to mammography and PSA tests, with an increase in proportion among less-educated people who attended the public health system. This result corroborates the goals for social equity established by the Health Protocol by improving efficiency and quality in the public health system responses.^5^

Besides the effort of the Brazilian public health system on monitoring Cancer Screening, such as the development of a computerized data entry system by the Brazilian Health Informatics Department (DATASUS) in partnership with the National Institute of Cancer (INCA), this system does not allow for the complete monitoring of actions related to cancer screening, early detection, diagnostic confirmation, and treatment initiation,^2^^,^^34^^,^^35^ thus requiring health surveys such as ISA-Capital to monitor the proportion of tests over time.

Being conducted with data from health surveys, the present study presents limitations inherent to this research design. ISA-Capital is conducted with self-reported data and thus is liable to errors in classifying participants' responses. Moreover, the analyses comprise the period until 2015, the most recent period with available data. Another limitation refers to the use of only one indicator (Years of study) to analyze social inequalities in access to preventive exams. However, Years of study are considered an excellent indicator and are used as the only Proxy in several surveys.

Despite these limitations, the present study included data from a population-based survey that provides representative information on the magnitude of the problem based on the most populous and one of the most unequal megalopoleis in Brazil.^15^^,^^22^ Moreover, by allowing us to measure information on health services, including different public and private services, ISA-Capital serves as an important tool to monitor inequalities and compare them with future studies.

## Conclusions

Access to cancer screening and early detection tests was higher among more educated individuals. Inequalities to access according to education level were more pronounced for mammography and PSA tests than for Pap smears, significantly decreasing throughout the study period (2003‒2015). Likewise, the coverage of these exams by the public health system significantly increased over time, especially for mammography and PSA tests, indicating the important role of the public health system in reducing health inequalities.

## Authors' contributions

Edige Felipe de Sousa Santos: Collaborated in the design of the article, methodology, data analysis and in writing of the article.

Camila Nascimento Monteiro: Collaborated in data analysis, writing and review of the article.

Diama Bhadra Vale: Collaborated in writing, review and editing the article.

Marília Louvison: Collaborated in writing and review the article.

Moisés Goldbaum: Collaborated in monitoring the analysis, writing and review of the article.

Chester Luiz Galvão Cesar: Collaborated in the methodology, monitoring of analyzes, supervision, writing and review of the article.

Marilisa Berti de Azevedo Barros: Collaborated in the design of the article, monitoring of analyzes, supervision, writing, review and editing the article.

## Conflicts of interest

The authors declare no conflicts of interest.
